# Stable Isotopes Indicate Population Structuring in the Southwest Atlantic Population of Right Whales (*Eubalaena australis*)

**DOI:** 10.1371/journal.pone.0090489

**Published:** 2014-03-05

**Authors:** Morgana Vighi, Asunción Borrell, Enrique A. Crespo, Larissa R. Oliveira, Paulo C. Simões-Lopes, Paulo A. C. Flores, Néstor A. García, Alejandro Aguilar

**Affiliations:** 1 Department of Animal Biology and IRBio, Faculty of Biology, University of Barcelona, Barcelona, Spain; 2 Laboratory of Marine Mammals, Centro Nacional Patagónico (CENPAT-CONICET), National University of Patagonia, Puerto Madryn, Argentina; 3 University of Vale do Rio dos Sinos (UNISINOS), São Leopoldo, Brazil; 4 Department of Ecology and Zoology, Center of Biological Sciences, Federal University of Santa Catarina,Trindade, 88010-970 - Florianopolis, Santa Catarina, - Brazil; 5 Centro Mamíferos Aquáticos - CMA (Centro Nacional de Pesquisa e Conservação de Mamíferos Aquáticos), ICMBio, MMA,, Jurerê Florianópolis, Santa Catarina, 88053-700, Brazil; 6 Study Group of Aquatic Mammals of Rio Grande do Sul (GEMARS), Imbé, Brazil; Institut Pluridisciplinaire Hubert Curien, France

## Abstract

From the early 17th century to the 1970s southern right whales, *Eubalaena australis*, were subject to intense exploitation along the Atlantic coast of South America. Catches along this coast recorded by whalers originally formed a continuum from Brazil to Tierra del Fuego. Nevertheless, the recovery of the population has apparently occurred fragmentarily, and with two main areas of concentration, one off southern Brazil (Santa Catarina) and another off central Argentina (Peninsula Valdés). This pattern suggests some level of heterogeneity amongst the population, which is apparently contradicted by records that traced individuals moving throughout the whole geographical extension covered by the species in the Southwest Atlantic. To test the hypothesis of the potential occurrence of discrete subpopulations exploiting specific habitats, we investigated N, C and O isotopic values in 125 bone samples obtained from whaling factories operating in the early 1970s in southern Brazil (n = 72) and from contemporary and more recent strandings occurring in central Argentina (n = 53). Results indicated significant differences between the two sampling areas, being δ^13^C and δ^18^O values significantly higher in samples from southern Brazil than in those from central Argentina. This variation was consistent with isotopic baselines from the two areas, indicating the occurrence of some level of structure in the Southwest Atlantic right whale population and equally that whales more likely feed in areas commonly thought to exclusively serve as nursing grounds. Results aim at reconsidering of the units currently used in the management of the southern right whale in the Southwest Atlantic Ocean. In the context of the current die-off affecting the species in Peninsula Valdés, these results also highlight the necessity to better understand movements of individuals and precisely identify their feeding areas.

## Introduction

Cetacean populations have been historically subject to different threats. Whaling, in particular, depleted many species which, due to their long life cycle and low demographic productivity, showed limited resilience. Consequently, many populations and several species of whales are currently catalogued by the International Union for the Conservation of Nature (IUCN) as endangered or critically endangered [1]. Since the International Whaling Commission put in place a protection policy and cut off commercial whaling, some populations have recovered although the pace of demographic increase has markedly varied with no evident reason for such heterogeneity.

An example of a strongly depleted population that is now recovering is the Southwest Atlantic population of the southern right whale (*Eubalaena australis*). Although the intensity of exploitation changed with time, the population was exploited almost continuously from 1602 to the mid1970s. Thus, the early 17^th^ century fishery conducted by Spanish and Portuguese coastal whalers [2] was substituted in the 18^th^ and 19^th^ centuries by a pelagic fishery dominated by open-boat British and American whalers [3] turning into a modern multinational operation [4], [5]. Exploitation of the species during the second half of the 19^th^ century and along the 20^th^ century was fragmentary and characterized by low levels of catches, undoubtedly due to the meagre state of the population. Thus, in the 20^th^ century about 800 whales were caught mostly in South Georgia between 1904 and 1931, a figure that represents only a small portion of the more than 200,000 whales caught during that period in these grounds [6]. Later on, between 1950 and 1973 about 350 whales were removed by coastal operations in Brazil [5] and about 1,300 were illegally caught by Soviet whalers in the late 1960s [7], [8]. From the late 1970s the protection of the species has been effective and the population has been recovering at an annual rate of about 7% [9], [10].

However, in the last years the population has experienced an enhanced mortality of calves that has raised concern about a potential curving of the recovery process [11]. The observed association between increased mortality and sea surface temperature oscillations [12] as well as the fact that the Peninsula Valdés right whales have fewer calves than expected in years of low krill abundance [11], may be suggestive that the population is approaching the carrying capacity of the ecosystem. However, this hypothesis would be apparently contradicted by the fact that, despite recorded catches by 19^th^ century whalers originally formed a continuum along the Southern American Atlantic coastline [3], the repopulation process has yet to complete filling empty spaces in the original distribution range. Thus, evidences for population increase only exist for two main areas of concentration, one off southern Brazil (around Santa Catarina) [13] and the other off central Argentina (Peninsula Valdés) [9], but not for the intermediate area (south of Uruguay and Mar del Plata), where the species appears to have been originally very abundant [14], [15], [16].

Such a fragmented pattern of recovery, which has also been observed elsewhere in other already well-recovered southern right whale populations [15], suggests some level of heterogeneity in the population which is apparently contradicted by movements of individuals recorded throughout the geographical range occupied by the species in the South Atlantic [17]. This apparent contradiction is not novel, since many examples are known among mysticetes of subpopulation units that never recovered after being extirpated by whaling [18]. Although mysticetes are highly mobile and migratory animals, individuals tend to return to particular feeding and breeding sites [19], [20] purportedly as a consequence of behavioural learning from their mothers during the first migration [21]. This matrilineal fidelity to specific breeding and foraging areas has been proposed to be acting as a limiting factor in the re-colonisation and population growth of the con-specific New Zealand population of southern right whales [22] and, indeed, genetic studies made in Peninsula Valdés showed that southern right whales concur in this behaviour and that they use non-homogeneous food sources [23]. In this context, and having whaling strongly depleted the Southwest Atlantic population of this species, it may be hypothesized that groups relying on different breeding or feeding grounds could have been kept isolated, and the lack of recovery in the central area of South America could be due to a loss of cultural memory, as proposed by Clapham *et al.* [18].

A sound understanding of the actual distribution and structure of a given population is essential for its management and conservation. For the Southwest Atlantic population of right whales this is particularly relevant because current management recognizes this to be a single stock, and therefore promotes integrated conservation actions along the Atlantic coast of South America [24]. When genetics do not delineate subpopulations, chemical markers and other tools, such as stable isotopes, may assist in achieving this goal [25], [26]. Stable isotope values in body tissues strongly correlate to the characteristics of the water masses in which feeding takes place and this property has made these markers a suitable tool to investigate diet, trophic ecology and migration in a variety of species, including marine mammals [27], [28], [29], [30].

Stable isotope values can be investigated in any body tissue, but bone has a slow isotopic turnover rate and therefore integrates a much wider temporal span than other more metabolically active tissues. This makes bone a tissue of choice for investigating long-term processes [31]. Bone is made up of two matrices, both of which are useful to investigate habitat use, pattern of migrations and dietary history of organisms. The organic matrix, mostly composed of collagen, is a complex structural protein that has been used to study nitrogen and carbon stable isotopes, the values of which are mainly related to trophic position and diet. The inorganic matrix, largely composed of carbonated hydroxyapatite, has been used to investigate variations in the oxygen stable isotopic value. This latter value correlates with characteristics of the water such as temperature and salinity and, as a consequence, is a good indicator of habitat use and migration patterns [29].

In this study we look into the nitrogen, carbon and oxygen stable isotopic values in bone samples of southern right whales from two separate locations on the southern coasts of South America, southern Brazil and central Argentina, to investigate potential structuring and isolation of subpopulations exploiting specific habitats.

## Materials and Methods

### Ethics Statement

All necessary permits were obtained to collect the samples for the described study, which complied with all relevant regulations. Samples from protected areas were collected with permits SISBIO 24429, 27927 and 17890 issued by the ‘Instituto Chico Mendes de Conservaçao da Biodiversidade’ in Brazil and with written provincial permits issued by the ‘Dirección de Fauna y Flora Silvestre, Subsecretaria de Recursos Naturales, Ministerio de Industria, Agricultura y Ganaderia’; and by the ‘Subsecretaría de Conservación y Áreas Protegidas, Secretaría de Turismo’, in Península Valdés, Argentina. No samples were donated or purchased, and no whales were killed for the purpose of this study. All the samples used for this research derived from animals stranded and naturally dead or from animals that were legally caught during the period of commercial exploitation.

### Sample collection and preparation

**Figure 1:**
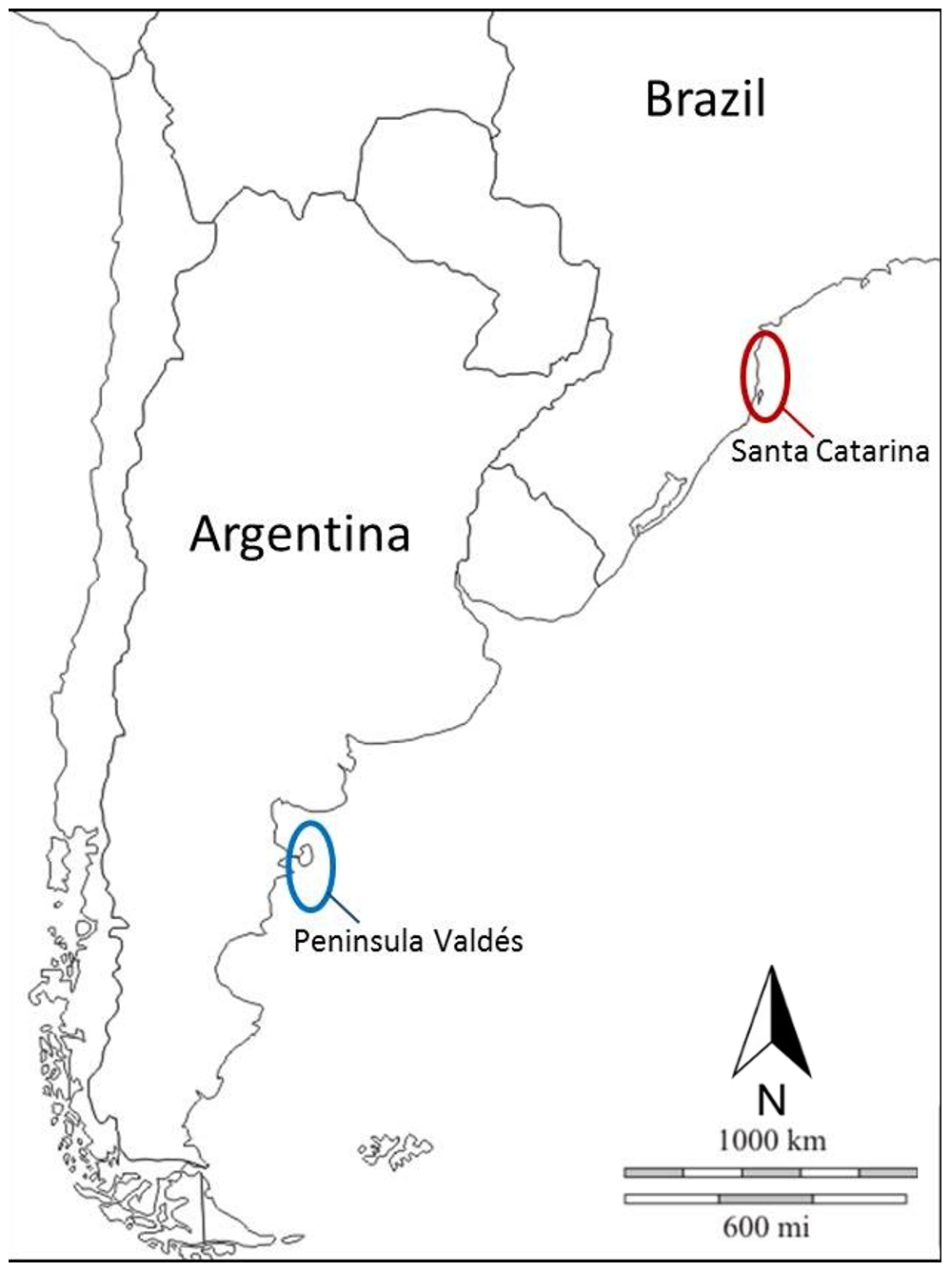
Sampling areas. Map of the Eastern coast of South America showing the two sampling areas: Peninsula Valdés, in central Argentina; and the area of Santa Catarina, in southern Brazil.

Bone samples were obtained either from individuals stranded or from disposal sites at the Southern Brazil whaling factories. In total, 125 samples were collected: 72 from the area off Santa Catarina, Brazil, and 53 from central Argentina, mainly from Peninsula Valdés, in the Chubut area (Figure 1). Samples from disposal sites were collected from sites located far apart in order to avoid the replicated sampling of a given individual; however, the occurrence of duplicates in the sample set cannot be totally excluded. All samples were modern (less than 50 years old), but information about the precise date of stranding or capture, as well as the sex or the age of the individuals involved, was not provided in most cases. Samples included different skeleton parts but in most cases they were parts of ribs, cranial bones, jaw bones and vertebrae.

The samples were preserved dry. For the analysis, a small subsample was ground to fine powder with mortar and pestle. Half of the powder was used for the analysis of carbon and nitrogen stable isotopes in the organic matrix, and half for that of oxygen stable isotopes in the inorganic matrix.

### Carbon and nitrogen stable isotope analysis

Because the depletion in ^13^C that occurs in lipids as compared with other constituents may affect the analytical results by decreasing the overall δ^13^C value in a sample [32], [33], prior to the analysis of carbon and nitrogen isotopes the bone powder was treated with a chloroform-methanol (2:1) solution to extract the lipophilic fraction [34]. Also, as the analysis of carbon and nitrogen stable isotopes was performed in the organic matrix of bone samples, and inorganic carbon is liable to affect the carbon isotopic value [35], [36], samples were decalcified with a 1 M solution of HCl.

After these treatments and subsequent drying, approximately 0.7 mg of each powdered sample was weighted in tin capsules, loaded, and combusted at 1000°C and analysed in a continuous flow stable isotope ratio mass spectrometer (Flash 1112 IRMS Delta C Series EA Thermo Finnigan) coupled with an elemental analyser.

Standards for ^13^C and ^15^N were the Vienna Pee Dee Belemnite (V-PDB) standard and atmospheric nitrogen, respectively. International isotope secondary standards of known ^13^C/^12^C and ^15^N/^14^N ratios, namely: polyethylene (IAEA CH7; δ^13^C = −31.8‰), sucrose (IAEA CH6; δ^13^C = −10.4‰), ammonium sulphate (IAEA N1; δ^15^N = +0.4‰ and IAEA N2; δ^15^N = +20.3‰), potassium nitrate (USGS 34; δ^15^N = −1,7‰), L-glutamic acid (USGS 40; δ^15^N = −4,6 ‰; δ^13^C = −26,2‰), and caffeine (IAEA 600; δ^15^N = 1,0‰; δ^13^C = −27,7‰) were used for calibration of δ^13^C and δ^15^N. The reference materials used for the analysis were selected based on previous calibration experiments performed on the same type of samples and in order to ensure an optimum range of reference values. All the reference materials used are distributed by the International Atomic Energy Agency (IAEA).

Results were expressed following the delta (δ) notation, in which the relative variations of stable isotope ratios are calculated as:
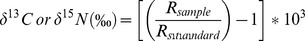



Where R is the heavy-to-light isotope ratio (^15^N/^14^N; ^13^C/^12^C) in the sample and in the reference standards certified by the International Atomic Energy Agency (IAEA, Vienna).

### Oxygen stable isotope analysis

Because the analysis of oxygen stable isotopes was performed in the inorganic matrix of the samples, the bone powder was treated with 30% hydrogen peroxide for 24 hours to remove the organic compounds. Samples were then rinsed carefully with milli-Q water and treated with 1 M calcium acetate/acetic acid buffer for another 24 hours to remove any diagenetic carbonate. Finally, samples were rinsed again repeating the same procedure and afterwards dried for 24 hours [28].

An average of 4 mg of each powdered sample was analysed using a Carbonate Kiel Device III carbonate preparation system (Thermo Electron-Dual Inlet) linked to a gas source mass spectrometer. Samples were dissolved in 100% phosphoric acid at 70°C with concurrent cryogenic trapping of CO_2_ and H_2_O. The CO_2_ was then admitted to the mass spectrometer for analysis. An internal standard calibrated with international standard NBS-19 was used. Precision was 1σ = ±0.05‰ for δ^18^O.

Values, reported in parts per thousand (‰), were calculated using the formula:
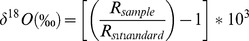



Where R is the heavy-to-light isotope ratio (^18^O/^16^O) and the standard is V–PDB. Because δ^18^O values in animal studies are more commonly presented relative to SMOW, to allow comparison with published data δ^18^O values were converted from V-PDB to SMOW using the following formula [28]:
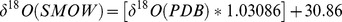



All the above analyses were conducted at the *Centres Científics i Tecnològics* (CCiT-UB) of the University of Barcelona.

### Statistical analysis

Results were tested for normality of distribution with a Kolmogorov-Smirnov test of goodness of fit and for homoscedasticity with a Levene test. The variability of the results and the presence of outliers were tested graphically through boxplots.

No analysis of the effect of age, sex or date of death in determining the isotopic values of the samples could be made because of lack of appropriate information.

The variances of the isotopic values from the two sampling areas did not satisfy the assumption of homoscedasticity, so the non-parametric test U of Mann-Whitney was used to detect differences between them. Also, a linear Discriminant Function Analysis with origin as grouping variable and δ^13^C, δ^15^N and δ^18^O as independent variables was used to establish the best combination of variables that distinguished the samples from the two areas and to determine the percentage of correct assignments to each of them.

A graphical comparison between the isotopic values of the two sampling areas was carried out with standard ellipses analysis (SEAc) of δ^13^C, δ^18^O and δ^15^N values performed using SIBER (Stable Isotope Bayesian Ellipses in R, [37]).

SPSS 20 and R-2.15.2 statistical software were used for all the above analysis.

## Results

Out of the 125 samples available, successful analyses could be conducted in 118 samples for nitrogen stable isotopes, 120 for carbon stable isotopes and 122 for oxygen stable isotopes.

**Figure 2:**
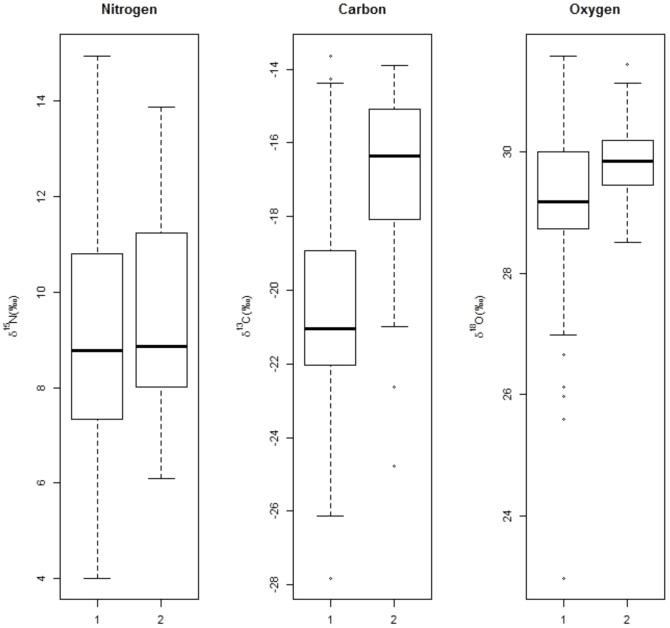
δ^13^C, δ^18^O and δ^15^N values distribution. Boxplots depicting the distribution of values obtained from the isotope analysis in the samples from the two sampling areas (1: central Argentina; 2: southern Brazil).

The analysis of the data distribution indicated an extremely high variability for the three elements (figure 2). δ^13^C ranged from −27.83‰ to −13.63‰ (mean±SD = −20.39±3.08‰); δ^15^N from 4.00‰ to 14.95‰ (mean±SD = 9.31±2.3‰) and δ^18^O from 22.97‰ to 31.59‰ (mean±SD = 29.54±1.17‰). Only one outlier was detected as being higher to 3*IQR (δ^18^O = 22.97‰). In order to check for potential errors in the processing of this sample, three aliquots from it were subsequently subject to independent analytical runs under the same laboratory conditions. The δ^18^O values obtained in the resulting four analytical runs were extremely variable, undoubtedly indicating the existence of heterogeneity within the sample. Taking this result into account, this outlier was eliminated from subsequent analysis. Excluding it, data obtained from the two sampling areas resulted normally distributed (Kolmogorov-Smirnov test) and with significantly different variances (Levene test, p<0.05).

Being the assumption of homoscedasticity unsatisfied, the comparison of mean δ^13^C, δ^15^N and δ^18^O between the two sampling areas was carried out through the non-parametric U of Mann-Whitney test. The results indicated that values for both elements were significantly lower (p<0.05) in the individuals from central Argentina (mean δ^13^C±SD = −20.39±3.08‰; mean δ^18^O±SD = 29.22±1.28‰) than in those from Brazil (mean δ^13^C±SD = −16.75±2.13‰; mean δ^18^O±SD = 29.87±0.62‰). No significant differences were found in δ^15^N between the two sampling areas.

The linear Discriminant Function Analysis correctly classified 77.6% of samples as Argentinian and 81.8% as Brazilian. Globally, the distinction between the two sampling areas based on their isotopic values was supported by the 80% of samples. The standard coefficients calculated for the discriminant function were 0.238 for δ^18^O; −0.7 for δ^15^N and 1.139 for δ^13^C. The statistics produced by this function for the Argentinian group of samples were: centroid (mean): −1; standard deviation: 1.112; and for the Brazilian group of samples: centroid (mean): 0.74; standard deviation: 0.909. The eigenvalue resulting from this function was 0.757, with an associated λ (Lambda of Wilks) value of 0.569, which was highly significant (p>0.001), indicating that the two sampling areas were statistically different.

**Figure 3:**
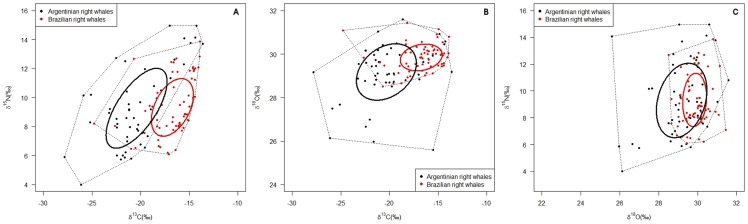
Standard ellipses analysis. Output graphs resulting from the standard ellipses analysis (SEAc) performed using SIBER (Stable Isotope Bayesian Ellipses in R, Jackson *et al*., 2011). Results from the analysis performed with δ^13^C and δ^15^N values (A), δ^13^C and δ^18^O values (B); and δ^18^O and δ^15^N values (C) are shown.

Standard Ellipses Analysis showed that the two sampling areas graphically differentiated mostly for δ^13^C and to a smaller extent for δ^18^O (figure 3). The overlapping area calculated from the plot of δ^13^C and δ^15^N values resulted to be the 6.08% of the area of the ellipse representing Argentinian samples and 9.95% of the area of the ellipse representing Brazilian samples. On the other hand, the overlapping area calculated from the δ^18^O-δ^13^C plot resulted to be the 10.37% of the area of the ellipse representing Argentinian samples, and the 40.72% of the ellipse representing Brazilian samples. Finally, as already announced by the U of Mann-Whitney test, δ^15^N values did not contribute to differentiating the samples coming from the two areas; the δ^15^N-δ^18^O plot shows how the ellipse obtained from the Brazilian samples is almost completely encompassed by the ellipse representing the Argentinian samples.

All results of the stable isotopes analyses described above are publicly available in the Supporting Information (Table S1).

## Discussion

Coincidentally with previous studies conducted in central Argentina [23], results obtained here for the three stable isotopes were highly variable in the two sampling areas. This large variability might be partially due to heterogeneities in the age, sex and death date of the individuals sampled and, in the particular case of our study, as well as to the type of bone used for the analysis. Unluckily, the actual influence on the isotopic values of these variables could not be established because adequate information from most individuals was not available. However, being whales highly mobile and thus able to cross long distances across the region, the high isotopic variability observed may be very likely reflecting the heterogeneous isoscapes of the Atlantic waters off South America, especially with regards to carbon and oxygen [38], [39], [40].

The various statistical tests highlighted a significant difference in mean δ^13^C and δ^18^O but not in δ^15^N between the two sampling areas. Being trophic position the main driver of δ^15^N variation [41], [42], the absence of significant distinction between areas is likely to indicate that all animals were feeding at similar trophic level. The diet of the southern right whale is mainly based on calanoid copepods and krill, particularly on species belonging to the genus *Euphasia* and *Munida*, which are generally situated at comparable trophic levels [43], [44], [45]. δ^13^C values, which in marine mammal tissues reflect those of the prey, vary with latitude, and show a strong correlation with the conditions of productivity prevailing in the area, appeared to be significantly higher in whales from Brazil than in those from central Argentina. This result is consistent with the baseline values available for the study areas as shown by the isoscapes delineated by McMahon *et al*. [40] for the Atlantic Ocean based on a meta-analysis of published plankton δ^13^C values. Thus, according to the isoscape, zooplankton δ^13^C baseline values in the Brazilian area appear slightly higher (around −20‰) than those in the Argentinian area (around −22‰). The difference between these values and our results is also consistent with the estimated diet-tissue discrimination factor available for bone of baleen wales, which has been estimated to be around +3‰ [46].

Similarly, the δ^18^O values showed a significant enrichment in the whales from southern Brazil as compared to those from central Argentina. Stable oxygen isotopes have rarely been used to investigate the migrations of marine vertebrates [47], [48] but δ^18^O, being strictly correlated with water salinity and temperature, can be a useful tracer for studying the use of habitat made by marine mammals in areas with defined environmental gradients. Again, comparing on a qualitative level the results here obtained with the available baselines for the sampling areas [39], [40], we found that the observed difference is consistent with proposed isoscapes for the South American Atlantic coast, which indicate slightly higher δ^18^O values in the Brazilian area than in the Argentinian one.

The standard ellipses analysis proved to be useful in graphically depicting the degree of isolation and overlap between the two sampling areas. Running the analysis with the pairwise combinations of the three isotopes was a good method for highlighting the contribution of every isotope in distinguishing the two sampling areas, and the calculation of the overlapping area numerically strengthened the results obtained.

Overall, individuals from southern Brazil were isotopically distinct from those from central Argentina. Being the carbon and oxygen isotopic composition of animal tissues mainly deriving from the food [49], a straightforward explanation for this difference is that individuals from the two areas exploit different feeding grounds. All previous evidences pointed to the Antarctic area as the main feeding destination for southwest Atlantic right whales as some individuals photo-identified in Peninsula Valdés had been re-sighted in South Georgia [50], a well-known feeding ground for the species [44], but the consistency of our results with the isotopic values around the sampling areas suggests that whales substantially feed also in their breeding grounds. Indeed, despite it is generally accepted that southern right whales primarily fast while they occupy lower latitudes, individuals have also been repeatedly observed feeding on plankton blooms, particularly at the end of the season [43], [45]. Feeding at lower latitudes has also been reported in other populations of southern right whales [51] as well in the northern right whale, *Eubalaena glacialis* [52] and would be further supported by frequent records in logbooks from open-boat whaling expeditions to the eastern coast of South America confirming the presence of “whale food” in the whaling grounds (unpublished data, extracted from 19^th^ century whalers logbooks), and the finding of floating faeces in Peninsula Valdés waters [11].

The isotopic difference between the individuals from the two sampling areas also might indicate that intermingling between the two areas is limited or non-existing, supporting the hypothesis of isolation between the right whales off central Argentina and southern Brazil. However, despite finding some level of genetic structuring, genetic results presented by Ott *et al*. [53] supported the hypothesis that whales from southern Brazil and central Argentina belong to the same population although recognizing that these individuals could mix on the feeding grounds with whales from other genetically distinct calving grounds (*e.g.* South Africa, [54]). Moreover, the isotopic result also appears to be contradicted by the re-sighting off Laguna (Brazil) of three mother-calf pairs that had previously been photo-identified in Peninsula Valdés [50]. Both the areas of Laguna (Brazil) and Peninsula Valdés (Argentina) are considered to be nursery grounds, even if calves appear more frequently in Peninsula Valdés [55]. To explain these occasional re-sightings it has been hypothesised that some females may not display a strict site fidelity and use therefore different nursery areas in different years or, else, that some females may move between the two areas in the year in which their calves are born [50]. However, records of long range movements of both the southern and northern right whales implying migrations over 4,000 km distances [56] confirm the high mobility of the species.

The absence of re-colonization of areas where the Southwest Atlantic right whales had been previously recorded and which now only appear to be or marginal use, strengthened by the isotopic differences found in the present study, seem to support the hypothesis that the recorded re-sightings are to be interpreted as random movements of the individuals. This hypothesis is also consistent with the recognized fidelity of females to the two current nursery grounds and the almost non-existing intermingling between the two areas. Another factor further supporting the differentiation between the two subpopulations of Southwest Atlantic right whales would be the occurrence of lesions caused by the attack of kelp gulls (*Larus dominicanus*), so far massively recorded in whales from central Argentina [57] and beginning only recently to be reported in whales from Brazil [58].

At the light of the above findings, the use of a single management unit for the conservation plans being developed for the Southwest Atlantic right whale and the application of an integrated approach for the species along the whole Atlantic coast of South America [24] should be reconsidered, as the population is likely to be structured into at least two units that exploit different feeding and nursery grounds. This distinction is particularly relevant not only from the demographic perspective, but also for the implementation of corrective measures since conservation threats are dissimilar in the two areas.

Thus, the main threats for the species in Southern Brazil are considered to be entanglements in gillnets, collisions due to the intense marine traffic and the whale-watching industry [11], [59]. Despite the number of right whales using the Brazilian nursery ground has been increasing since their exploitation ended in 1973 [60], the number of strandings in the last 20 years has been increasing [61], [62]. The higher rate of strandings is located in the Rio Grande do Sul area, which now appears to be only a migratory passage towards the nursery grounds situated further north, off Santa Catarina [63]. It is unclear whether the waters of Rio Grande do Sul have always been only a transit area or whether it ceased to be used as a nursery ground due to anthropogenic influence. If this latter were the case, regulation of shipping and fishing activities would be instrumental for promoting re-colonization and strengthening the conservation of this putative management unit [11].

On the other hand, the right whales calving in Central Argentina have recently experienced persistent high mortality events which affected 90% of the calves in their first year of life [11]. Causes adduced to explain the mortality include, among other potential factors, reduced food availability for adult females, bio-toxins and infectious diseases, as well as anthropogenic disturbance caused by whale watching, marine traffic and fisheries. However, harassment by kelp gulls appears to be central in the process, since in 2008 77% whales in the area presented wounds caused by those birds [64]. It has been suggested that kelp gull harassment is likely to affect behaviour and compromise calf survivorship [57] and therefore take a most relevant role in the whale mortality [65].

As a consequence of such dissimilar conservation scenarios, the two putative management units of Southwest Atlantic right whales would require independently focused conservation regulations and their biological parameters and demographic trends would have to be examined separately. Also, to deepen into the origin of the apparent demographic structuring of the population inhabiting the Atlantic waters of South America, the stable isotope analysis should be complemented with research based on other chemical and genetic markers as well as with historical reconstruction of past distribution, migration routes and localization of feeding grounds.

## Supporting Information

Table S1 **Stable isotope analysis results.** Table showing the list of samples with their corresponding sampling area and δ^13^C, δ^15^N and δ^18^O values.
(XLS)
