# Correction: Stable Isotopes Indicate Population Structuring in the Southwest Atlantic Population of Right Whales (*Eubalaena australis*)

**DOI:** 10.1371/journal.pone.0100024

**Published:** 2014-06-09

**Authors:** The PLOS ONE Staff

There is an error in the first name for the co-author listed as Alejandro Aguilar. The correct name for this co-author is Alex Aguilar.
